# Study on the Performance of a High-Speed Motor, Considering the Effect of Temperature on the Properties of High-Strength Non-Oriented Silicon Steel

**DOI:** 10.3390/ma17091936

**Published:** 2024-04-23

**Authors:** Yulin Li, Changhao Yan, Anqi Wang, Jun Li, Lubin Zeng, Ruilin Pei

**Affiliations:** 1 Department of Electric Engineering, Shenyang University of Technology, Shenyang 110870, China; l562098580@163.com (Y.L.);; 2 Suzhou Inn-Mag New Energy Ltd., Suzhou 215000, China

**Keywords:** high strength silicon steel, high speed permanent magnet synchronous motor, magnetic property, mechanical property, efficiency

## Abstract

Considering the high-speed and high power density technical specifications of new energy vehicle motors, there is a growing demand for rotor strength as motor peak speeds reach 20,000 r/min and beyond. The utilization of non-oriented silicon steel with a high yield strength in rotors has emerged as a promising approach to increase motor speed. However, the magnetic and mechanical properties of high-strength silicon steel under variable temperature conditions have not been fully explored, particularly in regards to their impact on motor torque, efficiency, and speed. This manuscript investigates the behavior of high-strength silicon steel before and after annealing and at different temperatures, analyzing its influence on high-speed motor performance. The validity and feasibility of this study are confirmed through prototype testing, providing a comprehensive reference for engineering design.

## 1. Introduction

Low-pollution vehicles have attracted great attention all over the world. In the trend of advocating for low-carbon initiatives, technologies related to new energy vehicles (EVs) are officially listed as major science and technology projects. The drive system of a new EV consists of three core components: motor, controller, and battery. As one of the three core components, the motor determines the dynamic performance of the new energy vehicle, and its innovation and development are particularly important. The technical capability of the motor and its control system directly affects the performance of the EV. Since 2004, the Toyota Prius has reported the performance of the electric vehicle drive motor, which has attracted the attention of engineers in the industry [1,2]. With more than ten years of development, high speed, high efficiency, and high power density have become the most important indicators for engineers [3,4,5]. In the future, these indicators will be even higher. The speed of the drive motors used in the new generation of electric vehicles developed by Xiaomi, Huawei, Tesla, and other manufacturers is almost above 18,000 r/min. Relying on traditional technology has been difficult when aiming to meet the performance requirements of the next generation of motors. Traditional methods used to improve the performance of motors mainly rely on the design of new topology structures, and the current research and application of new soft materials are insufficient.

For meeting the requirements of high-speed, high-efficiency, and high-stability motor performance in new EVs [6], it is necessary for non-oriented silicon steel to possess not only an excellent magnetic property, for achieving motor efficiency and energy savings, but also high mechanical strength, to safeguard the rotor against centrifugal forces during high-speed rotation. However, conventional strengthening methods tend to compromise the magnetic property of non-oriented silicon steel. Therefore, there is an urgent need to develop a new generation of high-performance non-oriented silicon steel with enhanced yield strength and exceptional magnetic property [7].

**Figure 1:**
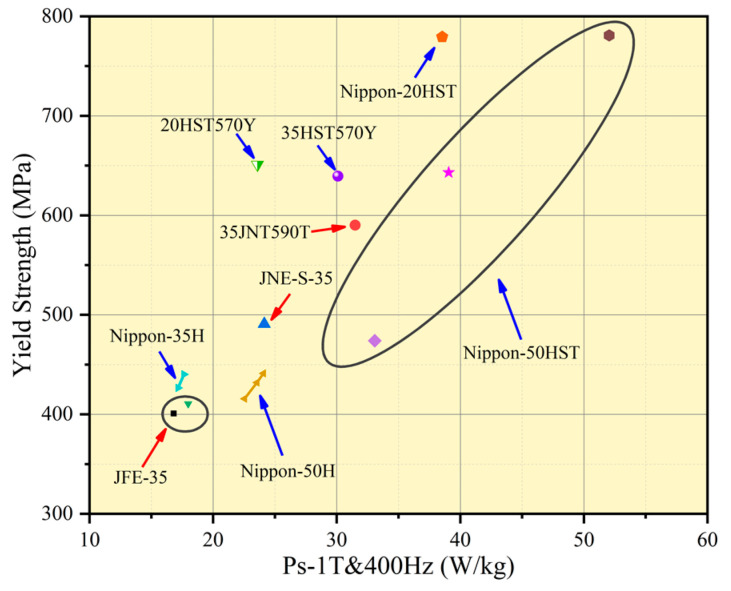
Properties of several high-strength non-oriented silicon steels [8,9,10].

In the 20th century, Japan’s JFE and Nippon Steel began to develop high-strength non-oriented silicon steel for new energy vehicle motors. Up to now, JFE and Nippon Steel have applied for invention patents based on preparation technology far more than other countries, and the performance of high-strength silicon steel products is in a leading position. The performance of some products is shown in Figure 1.

In 2008, Bao steel established a new generation of production lines for the preparation of high-grade, low-loss silicon steel, and began to develop high-performance, high-strength silicon steel for electric motors of new EVs. In 2022, Shou Gang Group announced that the world’s first dedicated line for high-performance silicon steel for new EVs began production. 20SW1200H, as its high-strength silicon steel, has extremely high yield strength and low iron loss, which provides new energy vehicles with greater torque, higher efficiency, and greater endurance. In order to prepare non-oriented silicon steel with a high yield strength and excellent magnetic property, it is necessary to combine different strengthening methods to improve the yield strength of the product based on the optimization of the design of the composition and process [11,12,13,14,15]. However, most studies have shown that the increase of strength often leads to the deterioration of magnetic property [16,17]. Therefore, selecting an appropriate strengthening method is crucial for the development and application of high-strength silicon steel. For most high-strength silicon steels, trace amounts of Ti, Nb, V, Zr, and other alloying elements are added on the basis of Si and Al elements to enhance the yield strength [18,19,20,21,22,23].

In view of the development of the motor industry for new EVs at home and abroad, the improvement of motor performance is inseparable from the properties of silicon steel. High-saturation magnetic flux density, high yield strength, and low iron loss are the main research directions. German scholars have proposed a kind of ultra-high-speed motor with an amorphous metal rotor [24] which makes use of a high mechanical strength and low loss of amorphous properties to achieve the properties of high power density and high efficiency. In 2020, German scholars continued to take high-silicon silicon steel as a breakthrough for motor optimization design and produced a prototype with a peak speed of 125,000 r/min [25], which verifies that high-silicon silicon steel is also a silicon steel with high yield strength.

In addition, Nippon Steel engineers applied high-strength silicon steel and ordinary silicon steel to a rotor core [26]. The torque property and efficiency of the motor under different flux barrier widths were compared. Although the iron loss of high-strength silicon steel is much higher than that of ordinary silicon steel, the efficiency of the motor was not greatly reduced. In 2020, high-strength silicon steel was used to make permanent magnet synchronous motors. Based on finite element simulations, the advantages and limitations of high-strength silicon steel replacing ordinary silicon steel were analyzed [27,28].

In summary, the preparation and application of high-strength silicon steel have been preliminarily explored; however, the research on the properties of high-strength silicon steel is not very comprehensive. This manuscript is a comprehensive study of this problem. In particular, when a motor is operating under high-temperature and high-frequency conditions, the properties of high-strength silicon steel can change and have an impact on the performance of the motor. These factors must be considered in the design of high-speed motors.

## 2. Experimental Design and Result Analysis

### 2.1. Design and Construction of Experiments

In the actual manufacturing of a motor, most engineers use the method of core annealing to reduce the core loss and further improve the efficiency of the motor. In the design of a rotor’s strength, engineers will also consider the S-N curve (S-N is a curve that shows the relationship between the strength and fatigue life of a sample under a specific cyclic stress. S-Stress/MPa, N-Fatigue life/cycles) and yield strength of silicon steel. In the future, for in the batch application of high-strength silicon steel, it is not only necessary to consider the risk problems caused by the reduction of its yield strength due to annealing, but also to consider the effect of the property changes of high-strength silicon steel at variable temperature on the performance of the motor’s torque, speed, and efficiency. In this manuscript, metallographic testing, Epstein testing, and tensile testing are used to explore the properties of high-strength silicon steel.

Metallographic testing is the study and analysis of the internal structure of metal materials. In this manuscript, the grain of high-strength silicon steel (Baosteel, Shanghai, China and Shougang, Beijing, China) is observed via Metallographic Inlay (LHM-1000, Lab Testing Technology, Shanghai, China) Metallographic Polishing (LAP-1E, Lab Testing Technology, Shanghai, China), and Metallographic Microscopy (M-45X, Lab Testing Technology, Shanghai, China). The properties of high-strength silicon steel are analyzed.

An Epstein frame is suitable for the testing of the magnetic property of silicon steel, and is one of the mainstream methods for the world’s major steel groups to test the performance of their silicon steel products. As a kind of non-oriented silicon steel, high-strength silicon steel is also suitable for magnetic property testing via this method. The sample size is 30 mm × 300 mm, based on the Epstein frame, and an AC (Alternating current) magnetic measuring system (TUNKIA-TD8520, Changsha, Hunan, China) is used to complete the testing (Temperature range: −70–200 °C).

**Figure 2:**
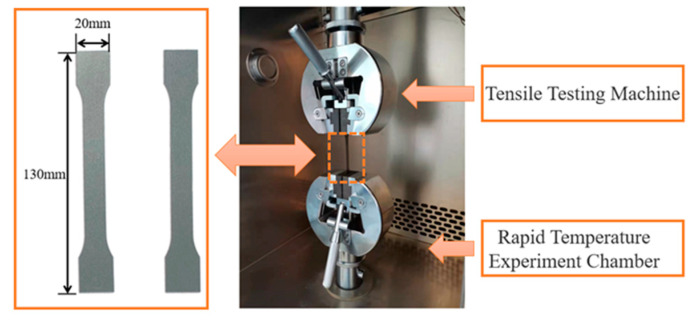
Principles of tensile testing.

According to GB/T 228.1-2010 and GB/T 228.2-2015, tensile tests of high-strength silicon steel at normal temperatures and high temperatures are completed. When a motor is working under load, the temperature of the iron core is generally between 80 °C and 120 °C (refer to the engineering design case of a high-speed motor). In this study, the test point of high temperature is 120 °C. The samples tested are standard dumbbell shapes with a size of 130 mm × 20 mm, and the standard distance of the deformation area is 50 mm. The test sample is placed in a chamber that can change its internal temperature, as shown in Figure 2.

### 2.2. Experimental Results and Performance Curve

The annealing temperature is the key parameter in the heat treatment process, and it varies based on the alloy system. Annealing involves heating the metal to a specific temperature and then cooling it at an appropriate rate to promote grain growth through recrystallization. Different soft magnetic materials require different annealing processes, while high-strength silicon steel can refer to conventional silicon steel for its annealing temperature. In this study, two different types of high-strength silicon steel are subjected to a 2 h annealing process at 800 °C to investigate their properties before and after annealing, as well as their changes at different temperatures. These results are compared with those of high-grade silicon steels of similar thickness.

#### 2.2.1. Metallographic Structure

**Figure 3:**
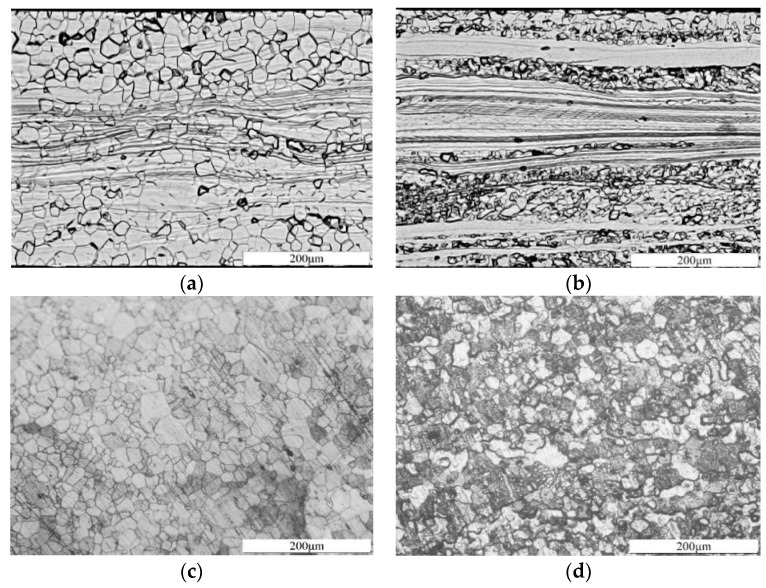
Metallographic structure of high-strength silicon steel: (**a**) 35AHS500 unannealed grain; (**b**) 35SWYS600 unannealed grain; (**c**) 35AHS500 annealed grain; (**d**) 35SWYS600 annealed grain.

The yield strength of ferritic materials is closely associated with the initiation, migration, and entanglement of dislocations. In the elongated and narrow deformed structures, a significant number of dislocations exist, which partially impedes the initiation and migration of new dislocations during material deformation, thereby enhancing material strength. To investigate the internal properties of high-strength silicon steel, metallographic observation tests are conducted using samples of 35AHS500 and 35SWYS600 as research subjects, as depicted in Figure 3.

In Figure 3a,b, it is evident that the microstructure of high-strength silicon steel features partial recrystallization prior to annealing, primarily consisting of equiaxed recrystallized grains and elongated deformed structures. Among them, the recrystallization ratio of 35AHS500 is approximately 70%, accompanied by a significant presence of dislocations within the deformed matrix. Conversely, the recrystallization ratio of 35SWYS600 amounts to about 30%, resulting in smaller grain size and higher yield strength. It can be observed that 35AHS500 high-strength silicon steel exhibits a typical Si-Al-Mn composition with minimal solid-solution-strengthening elements, such as Cr, Ni, and precipitated strengthening elements like Cu. Based on analysis of these two types of high-strength silicon steel, their enhanced yield strength compared to ordinary silicon steels can be attributed mainly to solid solution strengthening caused by the addition of Mn in conjunction with dislocation strengthening arising from the presence of elongated and narrow deformed structures.

The micrographs in Figure 3c,d depict the grain structures of two types of high-strength silicon steel following annealing treatment. The annealed grain exhibits a diminished prevalence of equiaxial recrystallization structures and elongated deformation structures, as compared to the unannealed grain. The grain size experiences a significant increase, accompanied by a substantial rise in the proportion of recrystallization. Grain size plays a crucial role in determining the magnetic and mechanical properties of silicon steel. To further investigate the impact of grain growth on the properties of high-strength silicon steel, this study conducts tests on its magnetic and mechanical properties before and after annealing.

#### 2.2.2. Electromagnetic Property

**Figure 4:**
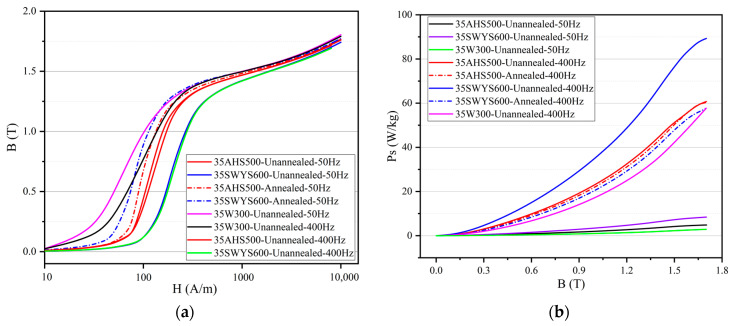
Magnetic property of high-strength silicon steel before and after annealing: (**a**) B-H curve (B–Magnetic flux density, H–magnetic field intensity); (**b**) Ps-B curve. (Ps–Iron loss per kilogram).

The B-H curve (50 Hz, 400 Hz) and the Ps-B curve (50 Hz, 400 Hz) of the high-strength silicon steel before and after annealing are compared in Figure 4.

In Figure 4a, following annealing, there is a noticeable increase in permeability and saturation magnetic flux density of high-strength silicon steel under the same magnetic field intensity. At a magnetic field intensity of 1000 A/m, the saturation magnetic flux density (B) increases from 1.469 T to 1.484 T for 35AHS500 and from 1.423 T to 1.498 T for 35SWYS600, respectively. The 35AHS500 exhibits a higher degree of recrystallization and relatively minimal change in grain size during annealing, resulting in a comparatively low variation in B before and after the annealing process. In general, annealing contributes to enhancing the permeability and B of silicon steel. At different frequencies, the slope of the B-H curve of silicon steel will also change slightly, which is caused by the different permeability at different frequencies [29].

The iron loss of silicon steel is proportional to the frequency. It can be seen from Figure 4b that under the same B value, the iron loss of silicon steel can be reduced by during the annealing process. When the frequency is 400 Hz, the iron loss of 35AHS500 decreases from 23.13 W/kg to 22 W/kg, and that of 35SWYS600 decreases from 35.13 W/kg to 20.53 W/kg. A comparison between these two types of high-strength silicon steel reveals that their magnetic properties are significantly improved after annealing and approach the typical values observed in high-grade silicon steel with the same thickness.

**Figure 5:**
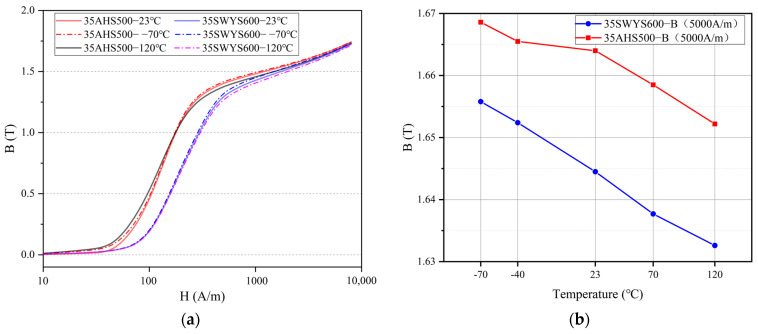
Magnetic property at variable temperature: (**a**) B-H curve; (**b**) The change of B_50_ (The value of B when the magnetic field intensity H is 5000 A/m).

The core is laminated with a structural adhesive (BEEP 6688, BEGINOR, Shanghai, China), which will fail at high annealing temperatures. As a result, the core is not eventually annealed. In Figure 5, the change of B of unannealed high-strength silicon steel at varying temperatures is compared. The results demonstrate a significant decrease in the typical value of B for high-strength silicon steel with increasing temperature, accompanied by a gradual reduction in the hysteresis loop area. This phenomenon can be attributed to the influence of temperature on electron and magnetic domain movement, wherein higher temperatures exert a greater impact on magnetic domains. As the temperature rises, the stability of magnetic domains decreases, transitioning from an ordered arrangement to a partially disordered configuration. Consequently, resistance within magnetic domains along the direction of the magnetic field increases with rising temperature, leading to increased magnetization difficulty and decreased B values. When the motor is working under load, this may cause its torque to decrease slightly.

**Figure 6:**
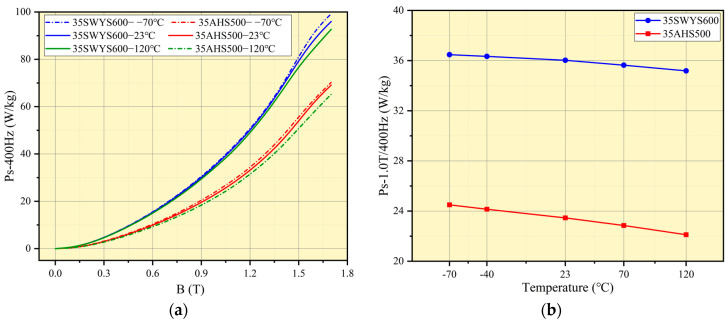
Ps-B curve at varying temperatures: (**a**) Ps-B curve; (**b**) The change of Ps at 1.0 T, 400 Hz.

The detailed results of the trend in iron loss for high-strength silicon steel at varying temperatures are shown in Figure 6. In Figure 6a, under the same magnetic field intensity, the iron loss of high-strength silicon steel decreases with increasing temperature, which is consistent with the property observed in ordinary non-oriented silicon steel. The increase in strength during preparation often leads to an increase in iron loss for high-strength silicon steel. The 35SWYS600 grade exhibits a higher yield strength and consequently shows a greater loss value during magnetization. When the motor is operating, it is common for the core temperature to exceed 80 °C, resulting in lower core losses and slightly improved motor efficiency.

For high-speed motors, the iron loss of silicon steel at high frequencies significantly impacts a motor’s efficiency, while the saturation magnetic flux density of silicon steel directly influences the motor’s torque. The optimization of the rotor using high-strength silicon steel can be further enhanced by refining the width of the flux barrier. Reducing this width effectively minimizes magnetic leakage and consequently enhances motor’s torque.

#### 2.2.3. Mechanical Property

**Figure 7:**
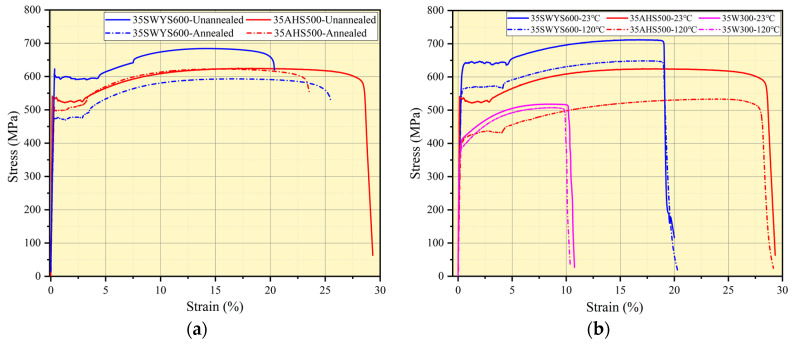
Stress-strain curves: (**a**) stress-strain curves before and after annealing; (**b**) stress-strain curves at varying temperatures.

To ensure sample consistency, each silicon steel is sampled under identical conditions. The mechanical properties of high-strength silicon steel are evaluated using dumbbell samples at various temperatures, as depicted in Figure 7.

The yield strength of different high-strength silicon steels exhibits a significant decrease before and after annealing, as depicted in Figure 7a. Specifically, the yield strength of 35AHS500 decreases from 524.99 MPa to 505 MPa after annealing, while the yield strength of 35SWYS600 decreases from 622.9 MPa to 488.4 MPa. The greater decline in the latter can be attributed to its lower proportion of recrystallization within the silicon steel structure. Notably, due to its smaller grain size, 35SWYS600 initially demonstrates higher yield strength. After the annealing process, complete recrystallization takes place, resulting in an increase in grain size that consequently leads to a more significant reduction in yield strength.

The yield strength of high-strength silicon steel exhibits a significant decrease with increasing temperature, as depicted in Figure 7b. At 23 °C, the yield strength of 35AHS500 is measured to be 523 MPa. However, at an elevated temperature of 120 °C, the yield strength decreases to 431.54 MPa, indicating a reduction of approximately 17.48%. Similarly, for 35SWYS600, the yield strength drops from 633.4 MPa to 564.4 MPa when transitioning from room temperature to 120 °C, resulting in a decrease of about 10.89%. In the case of high-grade silicon steel, such as 35W300, with identical thickness, its yield strength also diminishes from an initial value of 408.6 MPa to a final value of 380 MPa upon exposure to higher temperatures. Consequently, it is crucial to fully consider the impact of actual core temperature on the properties of high-strength silicon steel when designing the rotor strength for a high-speed motor. This consideration will not only enhance motor speed but also improve reliability and safety.

## 3. Finite Element Model and Performance Analysis of Motor

In practical engineering design, engineers anticipate motors to exhibit characteristics like “horses” that consume less energy and operate at higher speed, thereby possessing increased power density. Apart from motor size and winding current, rotor topology significantly influences motor performance. This study incorporates high-strength silicon steel in the flux barrier and rib of the integrated rotor, leveraging the properties of this material to enhance the motor’s torque and speed.

In this study, based on the complex operating conditions and performance requirements of the practical engineering applications of new energy vehicles, a high-speed permanent magnet synchronous vehicle motor is designed. Considering factors such as the torque of the motor and the linear speed of the rotor, the structure of the 8-pole -48 slot is finally selected, and the rotor topology is of type “UI” (Arrangement shape of permanent magnet) with a diameter of 121 mm. The design requirements of the motor are shown in Table 1, and the detailed structural parameters of the motor are listed.

### 3.1. Finite Element Model

**Figure 8:**
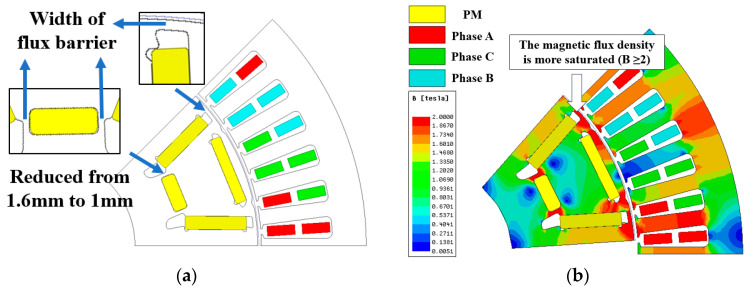
Finite element model of motor: (**a**) Change in the size of the flux barrier; (**b**) Magnetic flux density with load. (PM−Permanent Magnet).

The “UI” rotor structure has been designed, based on the fourth-generation model of Prius, to enhance power density and maximum speed. Through the finite element simulation analysis of its transient electromagnetic field, the performance of the motor with high-strength silicon steel is investigated. The three-phase winding is fed alternating current, adding the rotor to the “band” area to apply speed. Figure 8 shows the structure of the 1/8 finite element model of the motor in Ansys 19.2 (Canonsburg, PA, 15317, USA). The influence of the width of flux barrier and rib on the torque and speed of motor is mainly studied.

Under peak current conditions, the stator teeth exhibit a B value exceeding 1.7 T, while the flux barrier of the rotor reaches a saturated B value of 2 T. To enhance torque and minimize magnetic leakage in rotor structure design, it is imperative to minimize the width of both the flux barrier and rib. However, it should be noted that the yield strength of silicon steel imposes limitations on the width of these components. By employing high-strength silicon steel for rotor fabrication, we can elevate the allowable stress levels in rotor strength design, thereby increasing motor torque and speed.

**Figure 9:**
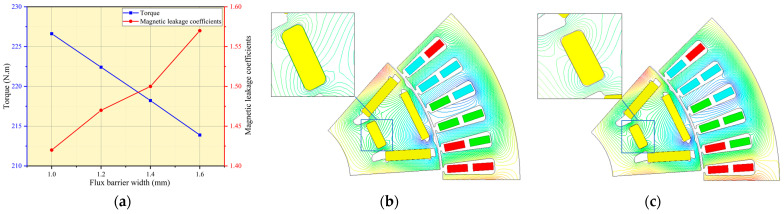
Performance of motor: (**a**) Torque and magnetic leakage coefficient at different flux barrier widths; (**b**) Flux lines of PM when flux barrier width 1 mm; (**c**) Flux lines of PM when flux barrier width 1.6 mm.

The magnetic leakage coefficient and torque of the motor are compared in Figure 9 for different widths of the flux barrier. When reducing the width of the flux barrier from 1.6 mm to 1 mm, there is a 5.95% increase in the maximum torque of the motor, which rises from 213.89 Nm to 226.62 Nm. Simultaneously, as the width of the flux barrier decreases, there is a decrease in the magnetic leakage coefficient from 1.57 to 1.42, resulting in an improved utilization rate of permanent magnet within the motor and subsequently increasing its torque.

### 3.2. Performance Analysis

The external characteristics and motor efficiency map reflect the distribution of motor efficiency under different operating conditions. By analyzing the efficiency map, we can determine the suitable working conditions for the motor based on its efficiency range. Based on the test data of high-strength silicon steel, the efficiency and speed of the motor are analyzed.

#### 3.2.1. Efficiency

**Figure 10:**
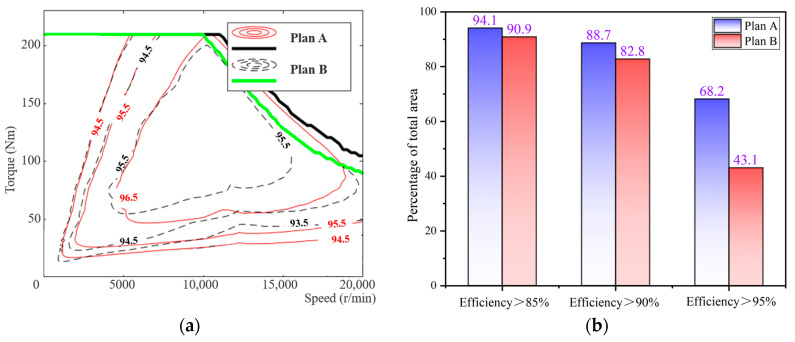
Efficiency analysis: (**a**) Efficiency comparison map; (**b**) Area ratio of efficiency.

The material for the stator and rotor of the motor is selected as 35AHS500. Based on high-temperature test data for high-strength silicon steel, plan A has a flux barrier’s width of 1 mm, while plan B has a width of 1.6 mm. The efficiency obtained through simulation calculation is illustrated in Figure 10.

The efficiency map comparison for the motor utilizing high-strength silicon steel before and after optimization is presented in Figure 10a. The computational results demonstrate that plan A achieves a maximum efficiency of 97.3%, while plan B only reaches 96.07%. Quantified indicators displayed in Figure 10b reveal that for plan A, the interval with an efficiency greater than 85% accounts for 94.1% of the total area, while intervals with efficiencies greater than 90% and 95% account for 88.7% and 68.2%, respectively.

Due to its preparation method, the iron loss of high-strength silicon steel is higher than that of ordinary silicon steel of the same thickness. However, after optimizing the flux barrier, the current required for the motor to output the same torque is significantly reduced. Reducing copper loss is also an effective means of improving motor efficiency, expanding the high-efficiency range and increasing maximum efficiency by 1.23%. Moreover, when the motor is in actual work, the iron loss of the core will be slightly reduced due to the high-temperature environment, thereby improving the operating efficiency of the motor.

#### 3.2.2. The Strength of the Rotor

The interference fit between the rotor and the shaft and the chamfer of the inner slot of the rotor will affect the stress of the rotor [30]. In this study, considering the feasibility of the application of high-strength silicon steel in high-speed motors and the practical feasibility of cutting, a 0.5 mm chamfer is added around the magnetic insulation bridge to reduce the stress concentration.

Centrifugal force is a main external load on the rotor of a high-speed motor. Through the statics analysis module of Ansys Workbench 19.2 (Canonsburg, PA, 15317, USA), the rotating speed load is applied and the static strength is simulated by finite element method. Since centrifugal force is a radial force, the length of the rotor in the axial direction does not affect the result. Therefore, the calculation time can be reduced by using a 2D plane model. In addition, the accuracy of the calculation can be improved by refining the mesh of the magnetic bridge region. Since the physical properties of silicon steel and structural steel (software default) are slightly different, new data are created based on the physical properties of high-strength silicon steel and permanent magnets. In the “Inertia” menu bar, the inner side of the rotor is loaded with speed, replacing the rotating shaft. As a purely Static external load, it is necessary to add “Rotational Velocity” in the “Static Structural” and set different rotational velocity values. As a result, the equivalent stress and strain corresponding to the rotational velocity can be obtained.

**Figure 11:**
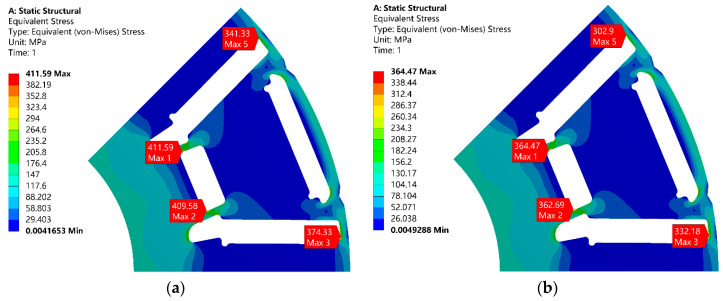
The simulation of centrifugal force.: (**a**) Plan A; (**b**) Plan B.

The rotors with different widths of the flux barrier and rib will bear different stresses when rotating at high speed. The rotational speed condition was set to 20,000 r/min, while considering a safety factor of 1.2 in engineering design. In the previous research work, the calculation results of binding, frictionless, and frictional contact modes between a permanent magnet and silicon steel sheet were compared. Under frictionless contact, the finite element calculation results of the model do not converge. However, under the binding contact, the calculation error is too large. Therefore, considering the glue between the silicon steel sheet and the permanent magnet, the real working state of the motor can be more accurately restored. The friction contact is set to the contact mode, and the friction coefficient is set to 0.15 (Generally between 0.1 and 0.2). Based on Ansys workbench 19.2, the calculation results of rotor strength are shown in Figure 11 below.

The red area represents the maximum stress experienced by the rotor during rotation. In plan A, when the flux barrier’s width is 1 mm, the rotor undergoes a maximum stress of 411.59 MPa. In plan B, with a flux barrier’s width of 1.6 mm, the maximum stress on the rotor is reduced to 364.47 MPa. Considering that the ordinary silicon steel used in this study exhibits a yield strength of only 380 MPa at high temperatures (120 °C), plan B appears more suitable. Decreasing the width of the flux barrier in plan A would result in increased stress on the rotor at similar speeds. By utilizing 35AHS500 for constructing the rotor, can we not only meet strength requirements but also enhance the motor’s torque and efficiency.

In this study, based on Plan A, the strength simulation of the rotor at different speeds was carried out to explore how much speed improvement can be obtained by the application of high-strength silicon steel to the motor. The results are shown in Table 2.

Under the same rotor topology, at a rotor speed of 18,000 r/min, the stress is calculated to be 355.21 MPa, which satisfies the design requirements for rotor strength considering the yield strength of ordinary silicon steel. By employing 35AHS500, the motor can achieve speeds up to 20,000 r/min, while using 35SWYS600 allows for a maximum speed of 24,000 r/min. Hence, incorporating high-strength silicon steel in high-speed motor design enables an increase in their maximum operating speeds. Additionally, it is crucial to consider the impact of actual iron core temperature on its yield strength to enhance safety measures alongside improving a motor’s performance.

## 4. Prototype and Test

**Figure 12:**
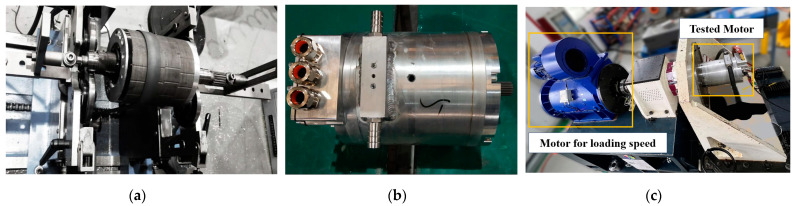
Prototype: (**a**) Dynamic balance test; (**b**) Motor; (**c**) 15,000 r/min test bench.

This research is aimed at the application background of new energy vehicles, referring to the test methods of <GB/T 1029-2021> [31] and <GB/T 18488.2-2015> [32] to test a motor based on high-strength silicon steel. The prototype of the optimized plan A is fabricated to further validate the accuracy of the simulation analysis, as depicted in Figure 12.

In the manufacturing method of the iron core, the method of wire cutting is chosen, which has the least impact on the magnetic properties of the silicon steel [33,34].

In the testing process, the type of measuring motor is a permanent magnet synchronous motor (PMW132L5P3/400 kW/545 Nm/20000 r/min), which is directly connected to the tested motor through the power connection shaft. The motor drive system is composed of a power module, inverter module, and filter module. For the torque sensor, the German “T40B” is selected, and the maximum torque can reach 1000 N.m, connected with the output shaft of the motor for loading the speed.

For the testing of the motor’s torque, speed, efficiency, and other characteristics, the load is applied to the tested motor. The test process starts with the maximum load and gradually decreases to the minimum load. At each load point, voltage, current, speed, and torque are measured, the efficiency and power factor of the motor are calculated, and the test process is automatically completed. For the temperature testing of the motor, its initial temperature $T_{0}$ and final temperature $T_{n}$ are recorded according to the temperature sensor placed in the stator windings. The formula $\Delta T=T_{n}-T_{0}$ is used to calculate the temperature rise.

**Figure 13:**
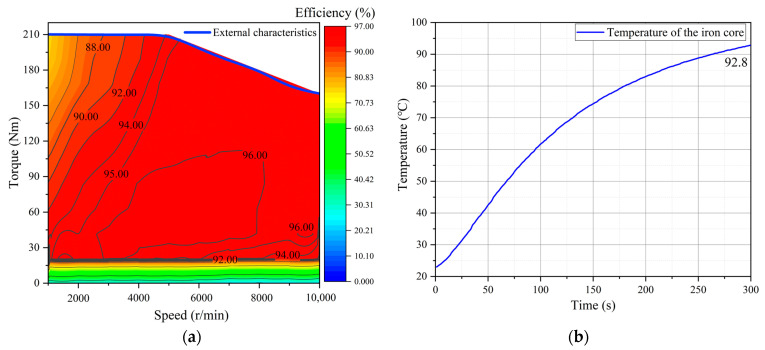
The test results of the prototype: (**a**) Efficiency map; (**b**) Temperature of the rotor.

Considering that the current capacity of the existing controller is limited to a maximum of 500 A, the peak current theoretical value for the high-speed motor designed in this study exceeds the testable range of the controller. Consequently, we evaluated the efficiency map of the motor at a current level of 450 A, torque ranging from 0 to 210 Nm, and speed varying between 0 and 10,000 r/min. The results obtained from these tests are presented in Figure 13.

The results depicted in Figure 13a demonstrate that when the current is 450 A, the high-strength silicon steel motor exhibits a torque exceeding 210 Nm, thereby achieving the targeted torque value. In terms of efficiency, the measured efficiency of the motor falls significantly short compared to simulation results. Specifically, there is a substantial reduction in the efficient area above 96%, as well as a decrease in the efficient area above 95%. Furthermore, there is a decline of 1% in maximum efficiency. As shown in Figure 13b, an infrared thermometer was employed to measure the temperature of the rotor, which reached an actual reading of 92.8 °C. The utilization of high-strength silicon steel for rotor construction meets all design requirements.

The test results show that the torque of the motor is significantly increased by reducing the width of the flux barrier, and the target torque value can be reached using only a 450 A current, which is 50 A less than the design value. The measured maximum efficiency data of the motor is decreased by 1% compared with the simulation, and the area greater than 95% efficiency is significantly reduced. In fact, when the motor is working under load, multi-physical fields, such as high temperature, high stress, and high frequency, will affect the magnetic properties of the silicon steel, thereby sacrificing the performance of the motor [35,36].

## 5. Discussion

For the discussion of the value obtained by the testing of high-strength silicon steel, the error mainly comes from the difference of the sample. For example, for B_50_ of high-strength silicon steel at variable temperature, to reduce the error, we tested the data three times and took the average value as the evaluation standard. From the test results, it was found that the error of Epstein testing on the value of magnetic flux density was less than 0.03 T. In addition, we selected the same grade of high-strength silicon steel, but samples were manufactured at different times, the B_50_ at 23 °C was 1.664 T, and the test data of the initial sample was 1.665 T. Therefore, this study is more expected to show the influence trend of temperature on the magnetic flux density of high-strength silicon steel, rather than specific data. Of course, this data also has a certain reference significance, as others may choose the same high-strength silicon steels to test, certainly also in the range of 1.663 T–1.665 T.

For the grade of silicon steel, although only two kinds of high-strength silicon steel with a thickness of 0.35 mm were selected in this study, their characteristics are applicable to all high-strength silicon steels. To improve the efficiency of the motor, the rotor was made of high-strength silicon steel, and the stator was made of 0.27 mm ordinary silicon steel. Therefore, the core was not annealed during the motor manufacturing process. In our opinion, if both the stator and rotor are applied with high-strength silicon steel, the stator could be annealed to further improve the efficiency of the motor. For this, the purpose of stator annealing is not only to eliminate the internal stress, but more importantly to reduce the hysteresis loss of high-strength silicon steel.

In the actual production process of a motor, it is also necessary to strictly optimize the assembly process of the motor, prevent any performance reduction caused by the process, and reduce the performance error between the simulation model and the measured prototype.

## 6. Conclusions

Based on high-strength silicon steel, this manuscript presents the design of a high-speed permanent magnet synchronous motor with a peak power of 180 kW and a peak speed of 20,000 r/min. A prototype based on high-strength silicon steel has been successfully manufactured, demonstrating that its performance is essentially in line with expectations. The main conclusions are as follows:

1. High-strength silicon steel exhibits higher yield strength due to its lower proportion of recrystallization structures compared to ordinary silicon steel. The recrystallization rate of 35AHS500 is about 70%, and that of 35SWYS600 is about 30%. For high-strength silicon steels, the yield strength is inversely proportional to the recrystallization rate. For high-strength silicon steel with a lower recrystallization rate, the iron loss can be reduced more significantly by annealing, but the yield strength can also be reduced.
2. With the increase of temperature, the yield strength of high-strength silicon steel with a higher recrystallization rate will decrease more significantly. The permeability is inversely proportional to temperature, and the iron loss is also reduced at a constant magnetic field strength (B). For high-strength silicon steel with a smaller grain size, the improvement effect of magnetic properties at high temperature is weaker.
3. In summary, there are many advantages in using high-strength silicon steel instead of traditional silicon steel in a rotor. On the one hand, the reduction of the width of the magnetic bridge can make the motor need less current to achieve the same torque, thus reducing the temperature rise of the motor. On the other hand, the maximum speed of the motor can be increased without changing the width of the magnetic bridge. However, for how much the speed can be increased, it is necessary to consider the yield strength of different high-strength silicon steels at high temperatures. For the motor in this study, after the application of high-strength silicon steel, its speed could be increased by at least 2000 r/min.

## Figures and Tables

**Table 1:** Parameters of motor.

Parameter	Value	Parameter	Value
Rated torque	210 Nm	Rated power	90 kW
Peak torque	90 Nm	Peak power	180 kW
Peak current	500 A	Width of flux barrier	1 mm
Outer diameter—Stator	180 mm	Outer diameter—Rotor	121 mm
Inner diameter—Stator	123 mm	Length of iron core	120 mm
Air gap length	1 mm	Diameter of copper wire	0.75 mm
Silicon steel—Stator	27AHV1400	Silicon steel—Rotor	35AHS500
Type of PM (Permanent magnet)	N48UH	Cooling method	Water

**Table 2:** Maximum allowable stress at different speeds.

Speed (r/min)	Maximum Allowable Stress (MPa)	Yield Strength-120 °C (MPa)
18,000	355.21	380 (35W300)
20,000	411.59	431.54 (35AHS500)
22,000	481.83	/
24,000	560.19	564.4 (35SWYS600)

## Data Availability

The data are not publicly available for privacy reasons. The data presented in this study are available from the corresponding author.
